# Ribosome RNA Profiling to Quantify Ovarian Development and Identify Sex in Fish

**DOI:** 10.1038/s41598-017-04327-y

**Published:** 2017-06-23

**Authors:** Zhi-Gang Shen, Hong Yao, Liang Guo, Xiao-Xia Li, Han-Ping Wang

**Affiliations:** Aquaculture Genetics and Breeding Laboratory, The Ohio State University South Centers, Piketon, Ohio 45661 USA

## Abstract

Terminologies of ovary development, by somewhat subjective describing and naming main changes of oocytes, have been criticized for confusing and inconsistency of terms and classifications, and the incurred consequences impede communication among researchers. In the present work, we developed regression between ovary development and three ribosome RNA (rRNA) indexes, namely 5S rRNA percent, 18S rRNA percent, and 5S–18S rRNA ratio, using close relationship between volume percent of primary growth stage oocytes or gonadosomatic index and rRNA content, demonstrating species-specific quantification of ovary development can be established in species with either synchronous and asynchronous oogenesis. This approach may be extended to any species with primary growth oocytes, e.g. anurans and reptiles, to predict maturity stages in females. We further confirmed that 5S rRNA percent and 5S/18S rRNA ratio can serve as markers to distinguish sexes unambiguously. A micro-invasive sampling method may be invented for non-lethal prediction of ovary development and sex because only a small amount of ovary sample (<50 mg) is needed for the approach established in the current work. Researchers who work with ovary RNA-seq in these taxa should realize that insufficient depletion of rRNA will probably lead to incorrect quantification of gene expression and inaccurate conclusions.

## Introduction

Sex differentiation, which is triggered by sex-determining factors, is to indicate the physical realization of the events in terms of ovarian or testicular development, including morphological, cellular, and molecular aspects^1–3^. Once primordial germ cells differentiated into either female or male germ cells, they will undergo gametogenesis, reach full maturation, and be ready for fertilizing, along with a series of complex molecular and endocrine changes. In the process of gametogenesis, oocytes and spermatocyte undergo significantly diverse molecular, cellular, and structural changes. Particularly, the oocytes increase size by hundreds of times and accumulate reserve substances, e.g. RNAs, proteins, lipids, carbohydrates, vitamins, and hormones, which are indispensable for proper development of the embryo^4, 5^. One of these molecules involved considerably in oogenesis is 5S ribosome RNA (5S rRNA). In the oocytes of some amphibian and teleost species, rDNA is amplified ≈1,000-fold and 5S rRNA and transfer RNA (tRNA) constituted more than 90% of the RNA content approximately at the end of primary growth or early cortical alveolus stage^6–9^.

The 5S rRNA was discovered as early as in 1963 and has been studied for RNA-protein interactions and used as a phylogenetic tool for several decades^10, 11^. However, its precise function remains unknown. Intriguingly, two different types of 5S rRNA genes with several sequence variations have been discovered in *Xenopus laevis*: one is only expressed in oocytes and the other in both somatic cells and gonads^12^. A similar pattern with two paralogue genes has been found in several fish species^6, 7, 13^. Specifically, only oocyte 5S rRNA accumulated in ovaries. The oocyte 5S rRNA undergoes considerable mobilization and decreases to baseline level when oocytes enter vitellogenesis and this massive amount of 5S rRNA is incorporated into the ribosomes in the amplified nucleoli^6, 14, 15^.

The dynamic feature of oocyte 5S rRNA during oogenesis had been studied in some fish species in the 1970s, e.g. *Tinca vulgaris* and *T*. *tinca* ^6, 7^. This interesting feature then has been overlooked. 5S rRNA in fish ovaries has not received attention, until recently, probably due to the booming development of Real-time PCR (RT-PCR) and RNA-seq and their application in biological research in the past decade. The RNA integrity number (RIN), an index for assigning integrity values to RNA measurement combining different features of rRNAs obtained from microcapillary electrophoresis, has served as a “standard” for RNA quality control^16^ since it has been established. The “abnormal” feature of 5S rRNA in developing ovaries, however, confused many researchers at the beginning because it has been difficult to obtain expected high RIN from developing ovaries, as we obtained from developed ovaries, different stages of testes and somatic tissue samples. The overwhelming accumulation of 5S rRNA in either gonochoristic developing ovaries or hermaphroditic sex-changing gonads in electrophoresis, has been reported or mentioned in several studies regarding RT-PCR and RNA-Seq^9, 13, 17–20^, indicating it is a general feature in gonads with primary growth oocytes in fish, reptiles and anurans. In addition, there are indications that ovary 5S rRNA profiling could be applied in the identification of sex, ovarian development, reproductive endocrine disruption^9, 13, 19^. Changes of 18S rRNA were also observed in fishes and amphibians^6, 21^.

However, how accurate are the rRNA features in sex identification and evaluation of ovary development? Does it work for species displaying asynchronous or synchronous ovaries? With these questions, plus our experience, the objectives of the present study are to: (1) develop an approach for sex identification and oogenesis stage determination in fish with asynchronous or synchronous ovaries using rRNA profiling and evaluate the accuracy; (2) alert researchers who are working on ovary transcriptomics in fish, anurans, and reptiles that incorrect depletion of rRNA, especially the mass accumulated 5S rRNA in developing ovary, may lead to misassembly and incorrect quantification of gene expression.

## Results

### rRNA profiling as powerful sex marker

**Figure 1:**
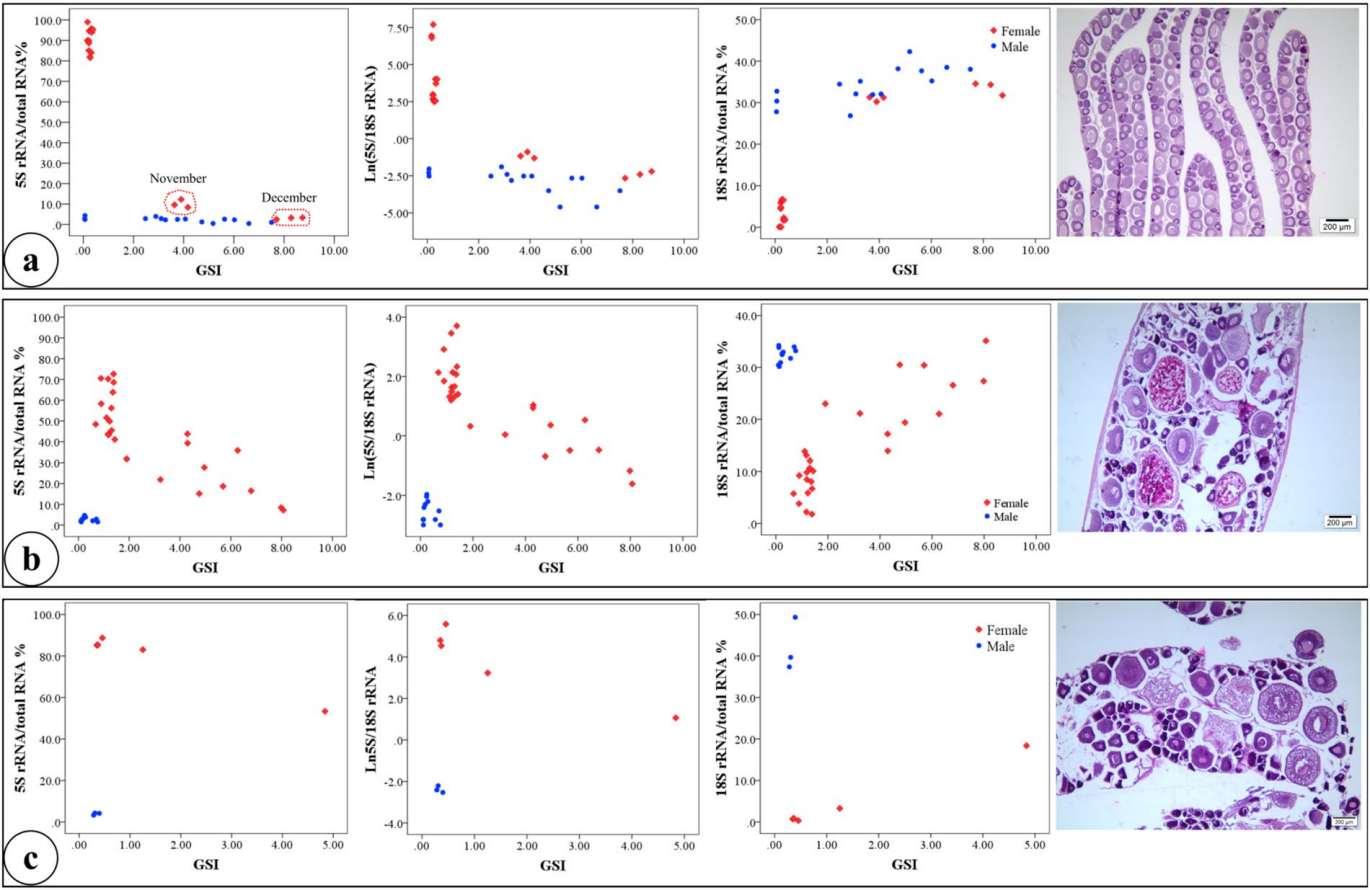
Ribosome RNA (rRNA) profiling in gonads as powerful sex marker in species displaying synchronous (yellow perch) and asynchronous (bluegill and largemouth bass) ovary development. Three indexes, percent of 5S rRNA relative to total RNA (5S rRNA/total RNA %), the natural logarithm of 5S/18S rRNA [Ln(5S/18S rRNA)], and percent of 18S rRNA relative to total RNA (18S rRNA/total RNA) were applied. (**a**) Yellow perch (n = 33), 5S rRNA/total RNA and 5S/18S rRNA can both distinguish females from males unambiguously from early ovary development up to four months (November point) before spawning season, but can’t later on; 18S rRNA/total RNA can distinguish females from males unambiguously only at early ovary development stages. (**b**) Bluegill (n = 37), 5S rRNA/total RNA and 5S/18S rRNA can both distinguish females from males unambiguously throughout the reproductive cycle; 18S rRNA/total RNA can distinguish females from males unambiguously only at early ovary development stages (about GSI < 4.00). (**c**) Largemouth bass (n = 7), three indexes can all distinguish females from males unambiguously at any ovary development stages. Histological images indicate synchronous development of oocytes in yellow perch, and asynchronous development in bluegill and largemouth bass. GSI, gonadosomatic index.

RNA integrity index (RIN) ranged between 8.4 to 10 for testis samples, and under detectable level (1) to 10 for ovary samples. No signs of RNA degradation were observed in any of the samples from either regular electrophoresis or chip run in Agilent 2100 Bioanalyzer. A distinctive band with peak size between 130–145 bp were observed without exception, in all samples including gonad, brain, and liver in five studied species, yellow perch, bluegill, largemouth bass, channel catfish, and Nile tilapia. The band, constituted up to 98.77% of the total RNA content in the transcriptome of ovaries as measured in the electropherograms (Fig. 1), was identified as 5S rRNA, as verified in previous work^9, 13, 19^. Pearson correlation analysis indicates the consistency between the two indexes 5S rRNA/total RNA and 5S/18S rRNA, with correlation coefficient r = 0.947 (P < 0.001), 0.975 (P < 0.001), 0.986 (P < 0.001) in yellow perch, bluegill, and largemouth bass, respectively.

In species displaying synchronous oocyte development, 5S rRNA/total RNA and 5S/18S rRNA indexes can both distinguish females from males unambiguously from early ovary development up to four months (November point) before spawning season (March for yellow perch), but not at the later month (Fig. 1a). The 18S rRNA/total RNA index can also distinguish sexes at early gonad development, but not during later ovary development (Fig. 1a).

In species with asynchronous oocyte development, 5S rRNA/total RNA and 5S/18S rRNA indexes can distinguish females from males unambiguously from early gonadal development up to gonadal maturation (Fig. 1b,c). The 18S rRNA/total RNA index can also be used to distinguish sexes at all gonad development stages in largemouth bass (Fig. 1c), while only at early development (GSI < 4) in bluegill (Fig. 1b). Histology images clearly demonstrated that synchronous development of oocytes occurs in yellow perch, and asynchronous development occurs in bluegill and largemouth bass (Fig. 1).

### Dynamic variation of rRNA: correlation with GSI and gonadal development

**Figure 2:**
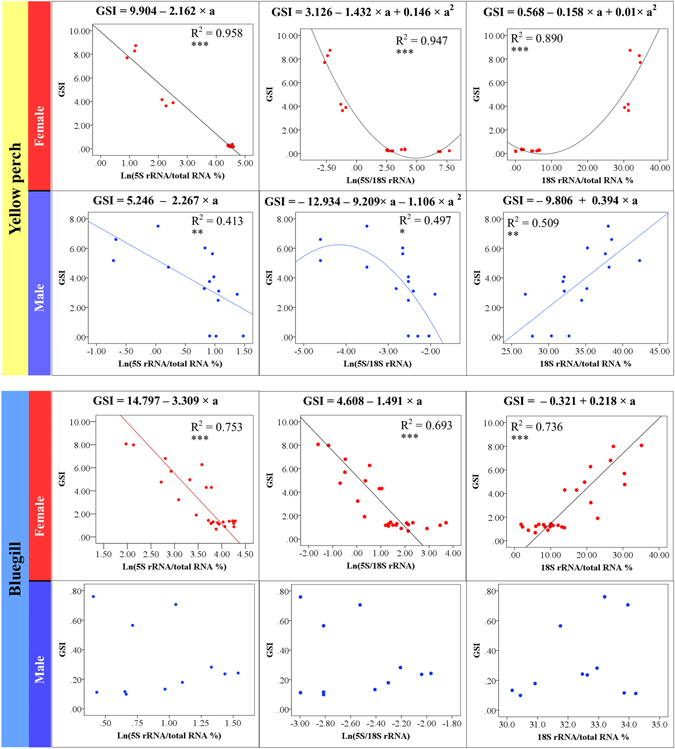
Regression of gonadosomatic index (GSI) and three ribosome RNA (rRNA) related indexes of females and males in fish species displaying synchronous (yellow perch) and asynchronous (bluegill) ovary development. Ln(5S rRNA/total RNA%), the natural logarithm of the percent of 5S rRNA relative to total RNA; Ln(5S/18S rRNA), the natural logarithm of the relative content of 5S rRNA to 18S rRNA; 18S rRNA/total RNA %, the percent of 18S rRNA relative to total RNA. The formula above each graph represents best curve fit generated by Curve Estimation in SPSS program (version 19). The Number of * under R square indicate the P value for formula estimation, with ***, **, * represent *P* < 0.001, *P* < 0.01, and *P* < 0.05, respectively.

**Figure 3:**
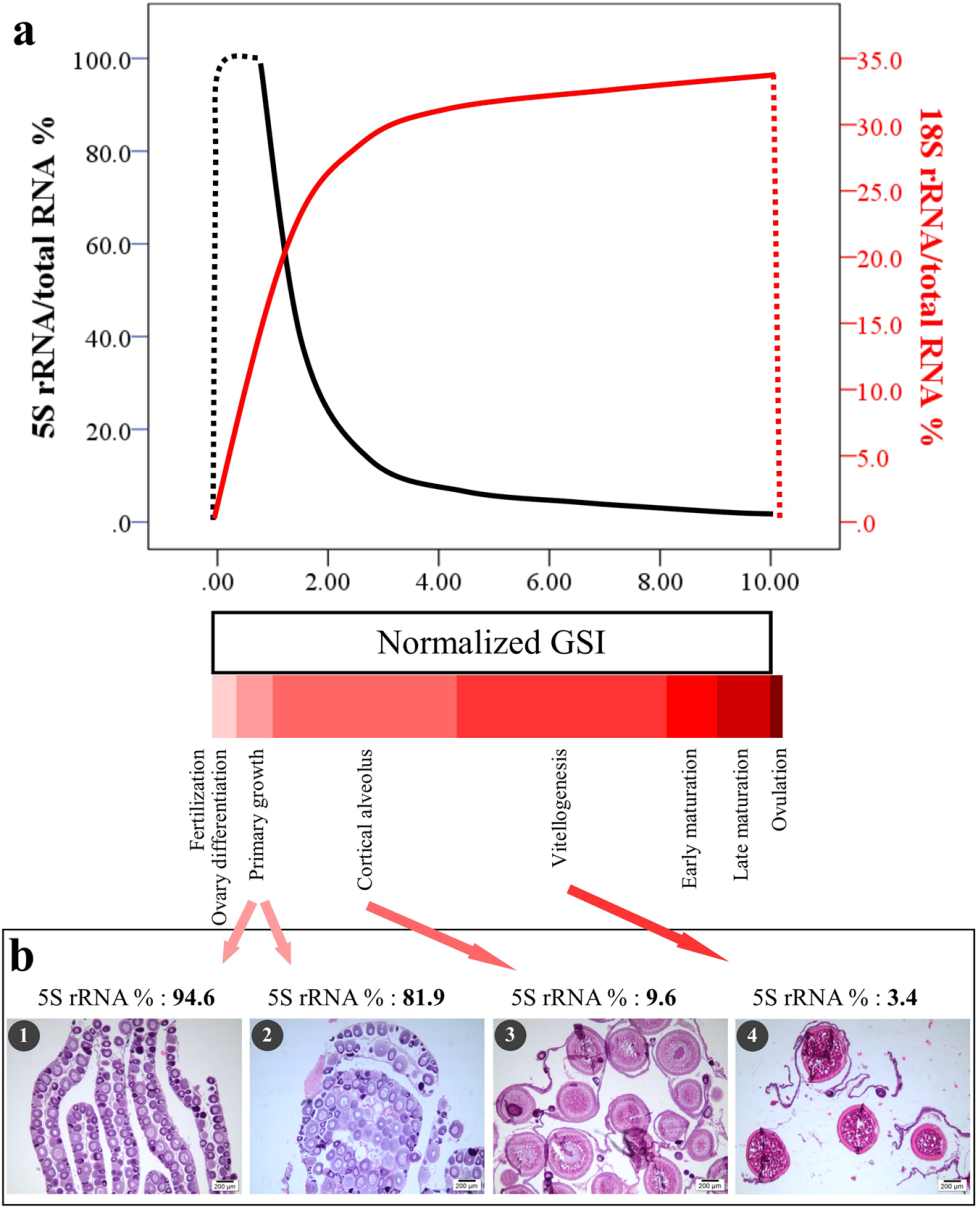
Simulation of dynamic changes of 5S and 18S rRNA percent in the ovary with oogenesis generated based on regression and some corresponding histology images in yellow perch. (**a**) Simulation of rRNA percent with normalized gonadosomatic index (GSI). The GSI on the X axis is normalized to 10 points scoring system. (**b1**) Primary growth stage, mainly composed of perinucleous oocytes; (**b2**) late primary growth stage, composed of perinucleous and a few cortical alveolus oocytes. (**b3**) Late cortical alveolus stage; (**b4**) vitellogenesis stage. Ovary development stages refer to histological observation in the current work and previous report^4^, and are not necessarily coordinate with the width of these blocks.

Pearson correlation analysis indicates a significant correlation between GSI and rRNA percent (5S and 18S rRNA) when female and male samples were analyzed separately. We then further examined whether a given rRNA content can be used to estimate the GSI. Results showed a good linear regression between Ln(5S rRNA/total RNA%) and GSI (R^2^ = 0.958), and quadratic regression between Ln(5S/18S rRNA) or 18S rRNA/total RNA % and GSI (R^2^ = 0.947, 0.890 respectively) in yellow perch females (all P < 0.001, Fig. 2). In bluegill females, three linear regressions were established with the highest R-square between Ln(5S rRNA/total RNA%) and GSI (R^2^ = 0.753), followed by between 18S rRNA/total RNA % and GSI (R^2^ = 0.736), and between Ln(5S/18S rRNA) and GSI (R^2^ = 0.693) (all P < 0.001, Fig. 2). Intriguingly, curve estimation also generated valid regression between rRNA content and GSI of the male in yellow perch, but not of the male in bluegill (Fig. 2). Based on the regression curve in yellow perch, we then generated a stimulation curve that including dynamic changes of both 5S and 18S rRNA percent as the ovary develops (Fig. 3).

**Figure 4:**
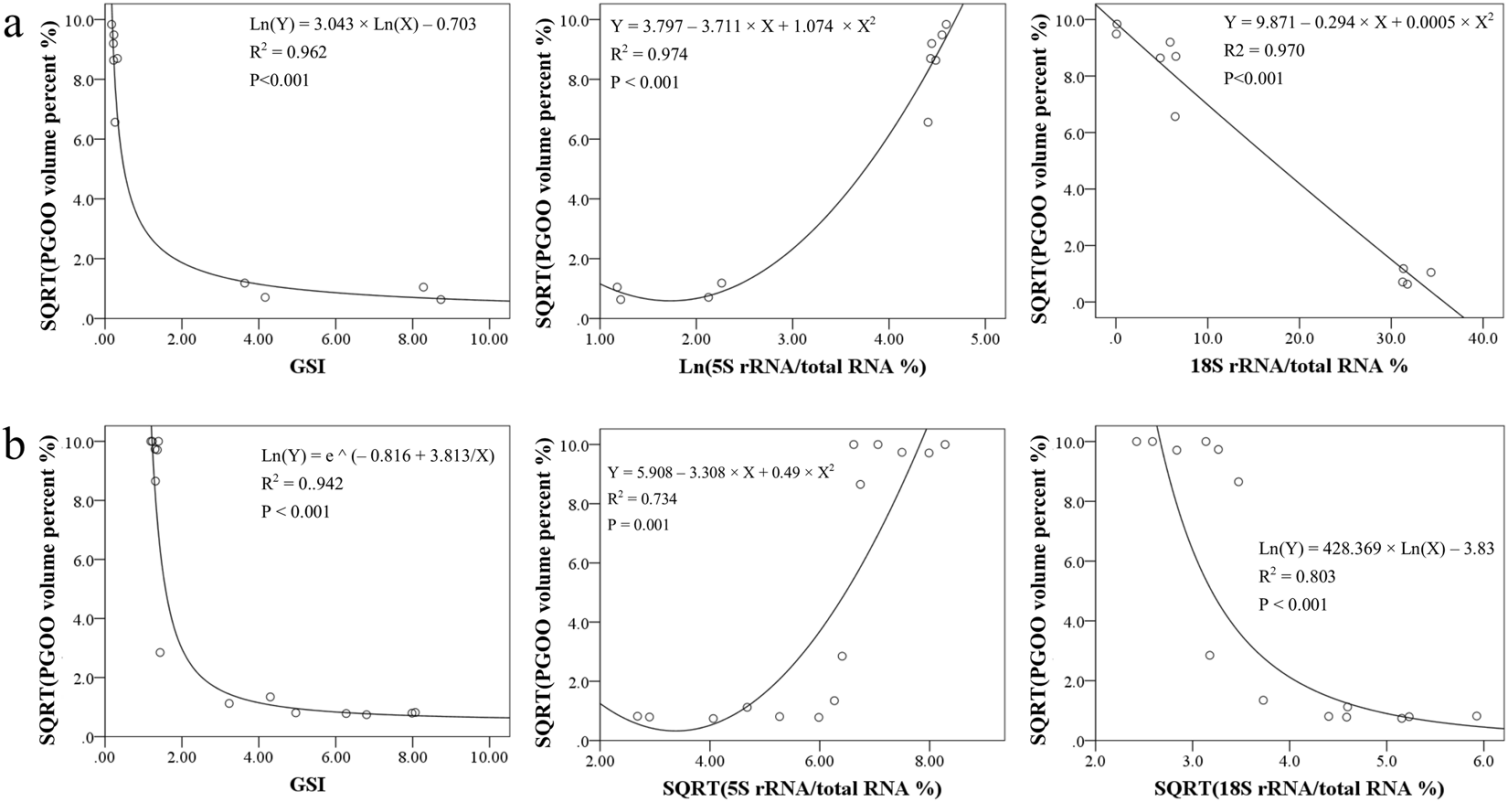
Regression between gonadosomatic index (GSI), 5S rRNA percent, or 18S rRNA percent and volume percent of primary growth stage oocytes relative to total volume of all oocytes. Ln, natural logarithm; e, natural constant; SQRT, square root. P, significance for formula estimation. Critical size points for primary growth oocytes are 184 and 174 µm for yellow perch (**a**, n = 10) and bluegill (**b**, n = 14) respectively, according to species-specific measurement in histological images and criteria by McMillan^4^.

The percentage was highest for 5S rRNA (98.77% for yellow perch and 68.58% for bluegill) and lowest for 18S rRNA (0.09% for yellow perch and 5.87% for bluegill) when ovaries are mainly composed of primary growth stage oocytes (PGOO, by volume, rather than by number); the opposite results were observed when ovaries were close to maturation (Fig. 3). Further analysis established good regression between volume percent of PGOO and GSI, 5S or 18S rRNA percent in both yellow perch and bluegill (Fig. 4).

Total RNA electropherograms of ovary samples in Channel catfish (6) and Nile tilapia (4), corresponding rRNA profiling and histological images can be found in Supplementary Figure S1.

### rRNA in somatic tissues

No signs of RNA degradation were observed in any of the samples from either regular electrophoresis or chip run in Agilent 2100 Bioanalyzer. RNA integrity number (RIN) indicates the high quality of RNA from both brain and liver samples (Table 1).

**Table 1:** Ribosome RNA (rRNA) percent of brain and liver in yellow perch and bluegill.

Species	rRNA	Brain	liver
Yellow perch	5S/18S	0.04 ± 0.03	0.08 ± 0.04***
*Perca flavescens*	5S/total %	1.19 ± 0.90	2.31 ± 1.06***
	18S/total %	33.49 ± 2.15	31.21 ± 2.66***
No.		30	30
RIN		9.16 ± 0.58 (7.6‒9.9)	9.44 ± 0.70 (8.1‒10)
Bluegill	5S/18S	0.04 ± 0.02	0.07 ± 0.03**
*Lepomis macrochirus*	5S/total %	1.72 ± 0.81	2.11 ± 0.86
	18S/total %	33.85 ± 2.89	31.92 ± 2.26*
No.		20	20
RIN		9.07 ± 0.54 (8.6‒9.7)	9.29 ± 0.62 (8.9‒9.9)

5S/18S, 5S–18S rRNA ratio; 5S/total %, 18S/total %, 5S or 18S rRNA percent relative to total RNA; *, **, and *** indicate significance *P* value < 0.05, <0.01, and <0.001 respectively, between brain and liver in each species. RIN, RNA integrity number generated by Agilent 2100 Bioanalyzer. Data are presented as mean ± Standard deviation.

There was no significance being detected in the three adopted indexes, 5S–18S rRNA ratio, percent of 5S rRNA, percent of 18S rRNA between sexes in either yellow perch or bluegill, or between species in either somatic tissue (brain and liver), while the 5S–18S rRNA ratio and 18S rRNA percent in brain tissue were significantly higher than liver in both species (Table 1).

**Figure 5:**
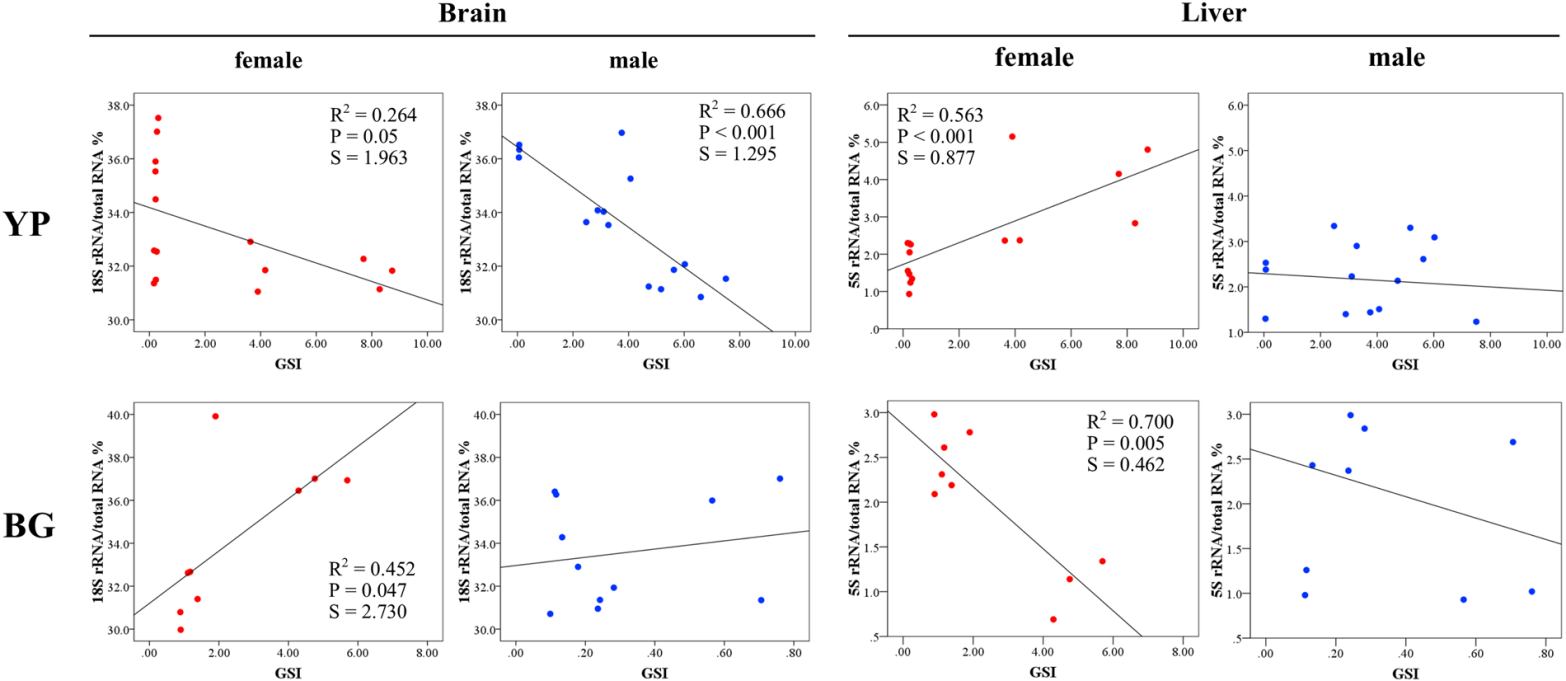
Dynamic variation of ribosome RNA (rRNA) with gonad development in brain and liver of yellow perch (YP) and bluegill (BG). GSI, gonadosomatic index. S, standard error of the estimate.

During gonad development, rRNA content also displayed interesting variation between yellow perch and bluegill. The 18S rRNA in the brain of yellow perch decreased during gonad development, which was more significant in males (Fig. 5). The 5S rRNA in the liver of female yellow perch increased during gonad development. The opposite tendency was observed in bluegill with regard to 18S rRNA in the brain or 5S rRNA in liver (Fig. 5).

## Discussion

The RIN has been considered the standard to evaluate total RNA quality, especially for studies that involve sequencing^16^. However, a few studies point out that it is not appropriate to analyze RNA obtained from ovaries due to the overwhelming accumulation of 5S rRNA in the developing ovaries^17, 18, 22, 23^. The mass accumulation and variation of oocyte 5S rRNA during oogenesis and two types of 5S rRNA had been well studied in anurans and fish in the 1970s^6, 7, 12, 24–26^; while the interesting features have been overlooked until recently (Kroupova *et al*.^9^; Diaz de Cerio O *et al*.^13^; Rojo-Bartolomé *et al*.^19^). Thanks to the technology combination of microfluidic chips, voltage-induced size separation in gel-filled channels, and laser-induced fluorescence detection on a miniaturized scale, Agilent 2100 bioanalyzer is capable of sizing and quantitation of RNA fragments in a high resolution^16, 27^.

### rRNA as a marker for sex identification

As beneficiaries of these abovementioned technologies, some researchers reported that the 5S/18S rRNA index, which is obtained from time corrected area of fragment peaks generated in bioanalyzer, can serve as a marker for sex identification in fish^19^. We found that in species with asynchronous oogenesis (ovaries consist of oocytes at all stages of development), either 5S rRNA percent or 5S/18S rRNA can be used to distinguish females from males unambiguously from very early stages of gonad development up to near maturation in bluegill and largemouth bass (Fig. 1b,c). While in species with synchronous oogenesis (ovaries consist of oocytes at the same stage of development), the two indexes can be used to distinguish sexes unambiguously if GSI is lower than 4 (Fig. 1a). We further found that, the percent of 18S rRNA, could also serve as a marker for sex identification in early gonad development stages in all three species studied (Fig. 1). Generally, sexes could be differentiated from appearance when GSI is about 4. Therefore, combining the results of 10 species with asynchronous oogenesis in a previous report^19^ and the present work with both synchronous and asynchronous spawners, we conclude that 5S rRNA percent or 5S/18S rRNA index in gonads can generally serve as an effective marker for sex identification in species displaying either synchronous or asynchronous oogenesis.

### rRNA profiling: quantification of ovary development

We established significant regression between three rRNA indexes, namely Ln(5S rRNA/total RNA%), Ln(5S/18S rRNA), or 18SrRNA/total RNA% and ovary development (represented by GSI). The trends of regression were similar between species with synchronous and asynchronous oogenesis for all these three indexes, while the regression estimate was higher for synchronous ovaries than asynchronous ovaries (Fig. 2). While GSI is a popular metric of maturity/reproductive condition, it may be an imprecise index for ovary development^28, 29^, especially when population underwent diverse growing conditions such as food, space, and competition. Therefore, we introduced an index called volume percent of PGOO (primary growth stage oocytes) and established significant regression with rRNA content (Fig. 4). It is recognized that an increase in size of oocytes is the most noteworthy manifestation of oogenesis^4^. Oocytes in primary growth stages undergo the most significant changes in terms of volume, specifically about 1000 fold^4, 30^. Meanwhile, during this stage the accumulation of nucleoli are thought to be associated with the massive production of rRNA^31^, which will be transformed and utilized for ovary maturation, e.g. enhancing protein synthesis^10^. Therefore, the process of ovary maturation involves the synthesis of proteins, which could be quantified by the changes of volume percent of PGOO and rRNA content. These results strongly suggest that species-specific quantification of ovary development can be established using a simple, consistent, and low-cost methodology, specifically ovary rRNA profiling (Fig. 3). The standard curve can be established through four main steps for rRNA profiling, including RNA isolation, electrophoresis in Agilent bioanalyzer, data collection and regression, and regular histological procedures (Fig. 3). In our lab, the first two steps of rRNA profiling for a hundred ovary samples can be completed in three days by one technician, while histological analysis (mounting, cutting, and staining) will take least two weeks for completion. An experienced technician is also required for photographing and analysis of histological images in order to deduce an acceptable conclusion. Agilent Bioanalyzer allows quick identification of absolute quantity through time corrected areas for 5S rRNA, 18S rRNA, and total RNA (Supplementary Figure S2). The time corrected areas for RNA can be copied to another program (e.g. Excel or SPSS) for further analysis and graphing. Once the species-specific (or taxon-specific) standard curve (100 samples including all ovary development stages will be more than enough) between rRNA index and ovary development stages has been established (Fig. 3), the laborious histological analysis could be largely replaced in related research fields in the future. In addition, the same 10-point scoring system for all species can be established so as to comparatively analyze oocyte development among species, taxon, or spawning strategies (synchronous ovaries, group-synchronous ovaries, and asynchronous ovaries). In addition, a micro-invasive sampling method may be invented for non-lethal identification of ovary development and sex, based on the approaches established in the current work, because only a small amount of ovary sample (<50 mg) is needed for RNA isolation and further analysis. Specifically, ovary development and sex could be identified without killing fish using rRNA profiling.

Traditionally, identification of the oogenesis stage in fish is dependent on a somewhat subjective description obtained from laborious histology and macroscopic observation^4, 32–34^. Researchers criticized that “ovarian terminology is confusing” three decades ago^35^; nevertheless, the criticism is still relevant^33, 34^. A major emphasis of a series of workshops on gonadal histology of fishes, including more than 30 papers, has been the development of terminology to describe the reproductive classification of fishes, due to the inconsistency and confusion^34^. Attempts to build up standardization and consistency into the reproductive classification in the past had not met researchers’ expectations, and the new classification system is still limited to describing and naming major phases during the fish reproductive cycle^34, 36^. In addition, a diverse group of scientists, including fishery biologists, fishery managers, ecologists, physiologists, toxicologists, environmental biologists, endocrinologists, development biologists, and aquaculturists all work with reproductive biology in both marine and freshwater fishes. Some of them need a unified terminology that addresses morphological and cellular changes, while others may be interested in physiological processes. Therefore, a consistent, simple, and quantitative terminology is urgently needed.

In addition, as the increasing of accessibility of RNA sequencing, a lot of studies are addressing molecular mechanisms involved in gonadal development, markers of egg quality and embryo viability, maternal genes, molecular pathways of sexual size dimorphism, etc. In such studies, obtaining enough RNA samples, identifying sex and/or gonadal development stage from a gonad sample of the same individual are frequently required. However, there is not enough gonad tissue for both RNA isolation and histological analysis, especially during early gonadal development^23^. While in these studies, electrophoresis analysis of RNA quality should be the indispensable procedure. Further, evaluation of fish fecundity, population sex ratio, and/or ovarian development are essential for various studies, such as fisheries, ecology, reproductive biology, sex determination and sex differentiation; traditionally it requires a considerable amount of samples for histological analysis^37–39^. Therefore, the approaches established for identification of sexes and quantification of ovary development in the current work, provide a low-cost, simple, and robust solution for these studies.

### rRNA variation in gonads and somatic tissues

In the present work, we found that 18S rRNA percent in ovaries underwent dramatic increase from 0.04% to about 33% (close to the level in somatic tissue) during ovary development. We also discovered that in species with synchronous oogenesis, both 5S and 18S rRNA in testes displayed similar trends of variation as the trends observed in the ovaries (Fig. 2). Interestingly, we observed significant changes of 18S rRNA in the brain and 5S rRNA in the liver during the progressing development of ovaries and testes (Fig. 5). The first finding was observed in *Xenopus laevis* long time ago^40^, while the other two have never been documented and the basics of dynamic variation of either 5S or 18S rRNA in gonad and somatic tissues are absolutely unknown.

Dynamic changes of ovary 5S rRNA had been observed more than four decades ago^6, 12, 24–26^, while the precise function of 5S rRNA in protein synthesis is not fully understood^11, 41^. Two types of 5S rRNA genes, designated as oocyte (or ovary) 5S rRNA and somatic 5S rRNA, have been discovered in many fish and anurans^6, 7, 12, 13^. Oocyte 5S rRNA is expressed in oocytes while somatic 5S rRNA is found in both somatic cells and gonads. Intriguingly, only oocyte 5S rRNA accumulated and then disappeared in ovaries^12^. Previous reports suggested that synthesis of 5S rRNA reached a peak at late primary growth or the early cortical alveolus stage (previtellogenesis stage), and was incorporated progressively into ribosomes when the oocytes enter vitellogenesis^9, 25^. Our data showed that 5S rRNA percent was the highest when ovaries only consist of primary growth stage oocytes in both fish species with synchronous and asynchronous oogenesis (Fig. 4), confirming that accumulation of 5S rRNA in ovaries reaches a maximum right before oocytes enter the cortical alveolus stage.

The small RNA molecules (5S rRNA and tRNA) accumulated in excess during the primary growth stage are stored as nucleoprotein particles, and are still present in the fertilized egg and in the developing embryo^42^. Fertilized eggs do not synthesize 5S rRNA and the capacity recovers later in development^43^. In light of the vital role of the ribosome in protein assembly, it could be hypothesized that the accumulation of 5S rRNA in small oocytes provides an important reservoir of material utilized during massive biosynthetic activities and is essential for early embryogenesis. It was hypothesized that investment toward the energetically demanding production of enough 5S rRNA during early oogenesis may be one of the prerequisites for oocyte secondary growth^19^. It is relevant because females may choose not to spawn or produce fewer eggs under unfavorable conditions, and the facts that increase of protein percent in egg matter results in an increase of fertilization rate and hatching success^44^. Intriguingly, the accumulation of 18S and 28S rRNA does not begin until the onset of vitellogenesis^21^. Therefore, 5S and 18S rRNA percent could potentially serve as supplemental markers for ovary development condition. It will also be necessary to study the discrete accumulation of 5S and 18S rRNA and the biological significance of their dynamic variation during oogenesis, ovary maturation, and early embryogenesis.

The dynamic changes of 5S and 18S rRNA during the development of testis probably associated with protein synthesis in spermatogenesis as in oogenesis. As displayed in Fig. 2, the regression between rRNA content and testicular development is significant, though the curve is not as good as in ovaries. Interestingly, the variation pattern of the curves are similar for testes and ovaries, indicating the importance of rRNA accumulation in reproductive success for both females and males. Though the spermatocytes do not undergo such dramatic changes as for oocytes, the variation of rRNA along with the testicular development deserves further investigation.

Changes of rRNA in brain and liver tissue as the gonads develop suggest their participation in oogenesis and spermatogenesis. The *18S rRNA* gene, which has been used as an internal control for gene expression normalization more than a thousand times (Thellin *et al*. 1999; Bustin 2000; Schmittgen and Zakrajsek 2000; Radonić *et al*. 2004; Zhang and Hu 2007), should not be used as an internal control for any gene expression studies because of its variation in gonadal and somatic tissues.

### 5S rRNA accumulation: problem in RNA-seq

The application of RNA-seq for transcriptomics analysis is booming in recent years. The ovary is one the important organs of interest regarding many research fields, e.g., genes and pathway networks involved in sex determination and sex differentiation, sex inversion of hermaphrodite, egg quality and fecundity, oogenesis, and molecular marker discovery. It has to be mentioned that the massive amount of ovary type 5S rRNA, which is unwanted and problematic RNA for in-depth transcriptomics analyses, will lead to insufficient coverage of transcripts of interest^45, 46^, if they are not correctly depleted, as we experienced for data generated from developing/resting ovaries. In one of our recent work^47^, the coverage (expressed as a number of raw reads) for samples from resting ovary (right after spawning) was only about a half for samples from testis or muscle (see Table S1). In an application report by Agilent Technologies (5989-1086EN) mentioned that column purification would remove the peak for small RNAs^48^. Mittelholzer *et al*.^49^ also proposed that a simple replacement in the isolation method from isopropanol precipitation to spin column purification would remove large peaks of 5S rRNA and tRNA. However, several recent works^19, 20, 47^ and the current work using different commercial spin column kits for RNA purification did not remove the peak for small RNAs, probably because the massive amount of small RNAs exceed the capacity of these commercial columns. Insufficient coverage will lead to a series of downstream issue in the transcriptomic analysis, e.g., misassembly, incorrect quantification of gene expression, and inaccurate conclusion^46, 50–52^, especially when whole genome sequence is not available. Popular methods to deplete problematic RNAs involve hybridizing the RNAs with biotinylated LNA probes as in several commercial kits or hybridizing the cDNA library to biotinylated DNA probes^53^. These probes usually target at specific rRNA without customization in commercial rRNA removal kits and suggest using the highest quality RNA possible. As far as we know, only a small proportion of scientists have realized the massive unwanted 5S rRNA in developing/resting ovaries of fish, anurans, and reptiles and the subsequent problem in transcriptomics analysis; and there is no commercial kit targeting this issue yet.

In conclusion, the present work found that rRNA profiling could serve as a low-cost, simple, and robust approach for sex identification in fish with synchronous and asynchronous oogenesis, and may be extended for reptiles and anurans. Species-specific quantification of ovary development, specifically, the standard curve between rRNA profiling [Ln(5S rRNA/total RNA%), Ln(5S/18S rRNA), or 18SrRNA/total RNA%] and ovary development stages can be established and utilized as a reference for future prediction of ovary maturity in females. Especially, significant regression between rRNA content and volume percent of PGOO or GSI suggest that ovary development/maturity could be quantified. The confusing and inconsistency of terminology for ovary development may be resolved when this approach is utilized in a considerate amount of species. Meanwhile, laborious histology work can be largely reduced. The regression between testicular development and rRNA content deserves further investigation because the similar regression pattern in testes and ovaries indicate the importance of rRNA accumulation in reproductive success in both females and males. Researchers who work with ovary RNA-seq should realize that insufficient depletion of rRNA will probably lead to incorrect quantification of gene expression and inaccurate conclusions.

## Methods

### Sample collection

This study and all experimental procedures involving animals were performed according to the protocol approved by the Ohio State University Institutional Animal Care and Use Committee.

Five major aquaculture species were used for this study, with emphasis on two species: yellow perch (*Perca flavescens*) with synchronous oocyte development, and bluegill (*Lepomis macrochirus*) with asynchronous oocyte development. Yellow perch, bluegill, and Nile tilapia (*Oreochromis niloticus*) were obtained from the breeding center of The Ohio State University South Centers. Largemouth bass (*Micropterus salmoides*) and Channel catfish (*Ictalurus punctatus*) were purchased from a local commercial fish hatchery. Following anesthetization using MS-222 and measurement of body weight, total length, and gonad weight, gonads of all individuals were dissected and divided into two parts. One part of the gonad was embedded in RNAlater® Stabilization Solution (Ambion, Life Technologies, U.S.A.) and kept at 4 °C overnight and then stored at −80 °C until further analysis. The other part was fixed in Prefer (Anatech LTD., MI, U.S.A.) for histological analysis. Goandosomatic index (GSI) is expressed as percent of gonad weight relative to total body weight. Livers and brains were also collected and stored in RNAlater for further analysis.

### RNA extraction and rRNA profiling

Total RNA was extracted from 30–100 mg of tissue using TRIzol® Reagent with the PureLink® RNA Mini Kit (Ambion, Life Technologies, U.S.A.) following the manufacturer instructions. Isolated RNA samples were evaluated by Nanodrop 1000 (Thermo Fisher Scientific Inc., U.S.A.) for concentration and by Agilent RNA 6000 Nano Kit Bioanalyzer (Agilent Technologies, USA) for capillary electrophoresis. Generated data were analyzed using Agilent 2100 Expert (free version B.02.08.SI648). The electropherograms generated in Agilent 2100 Bioanalyzer permit fast identification and quantification of 5S, 18S and 28S rRNA, and further comparative analysis. In the current case, as shown in Supplementary Figure S2, the peak size of 5S rRNA ranges from 131 to 144 nucleotide which can be easily differentiated from tRNA (ranges from 99 to 110 nucleotide) and 5.8 SrRNA (ranges from 164 to 168). The three small-sized RNA can be distinguished unambiguously in all samples though the peak size deviated from the real length (~120, ~150, and 75–95 for 5S rRNA, 5.8S rRNA, and tRNA, respectively) due to the conversion from time to size in the electropherogram. The time corrected area of each peak was used to calculate 5S rRNA/total RNA, 18S rRNA/total RNA, and 5S–18S rRNA ratio, without the interference of RNA concentration when comparing samples. Time corrected area for the marker was removed before further analysis. When time corrected area of either 5S or 18S rRNA was below the lower limit of detection of the machine (0.2), an estimation was given to each sample instead of 0. A full description of rRNA profiling can be found in Supplementary Figure S2.

### Histological analysis

Gonad tissues were transferred into 70% ethanol, gradually dehydrated, cleared, embedded in paraffin, cut at 5–7 µm, stained with hematoxylin, counterstained with eosin, and mounted, following routine histological procedures^54–56^ with extended time for ovary samples. Tissue slices were examined and photographed under a light microscope with an imaging system (Olympus cellSens Standard 1.13, U.S.A.). At least four cuttings from two slices were analyzed from each individual. Oocytes were considered as oblate ellipsoid and volumes were calculated using the following formula: Oocyte volume = πab^2^/8, where a and b are long and short axises of oocytes respectively. Each oocyte which displayed a nucleolus was measured and counted from each cutting. Critical size points (long axis) for primary growth oocytes are 184 and 174 µm for yellow perch and bluegill respectively, according to species-specific measurement in histological images and criteria by McMillan^4^.

### Statistical analysis

Data were analyzed using the SPSS program version 19. The correlation was estimated using two-tailed Pearson correlation. Best regression was obtained through comparing models generated using Curve Estimation. Data sets were checked for variance homogeneity and normality before further analysis. A t-test was applied to analyze the difference of rRNA percent in brain and liver. Significance was indicated at a 5% level (*P* < 0.05).

## Electronic supplementary material

Dataset 1
