# Supplementary material for: Ribosome RNA Profiling to Quantify Ovarian Development and Identify Sex in Fish
Source: Sci Rep. 2017 Jun 23;7:4196. doi: 10.1038/s41598-017-04327-y (PMC5482860; doi:10.1038/s41598-017-04327-y)
Supplement: Supplementary file 1 — Dataset 1 [file 41598_2017_4327_MOESM1_ESM.docx]

Ribosome RNA Profiling to Quantify Ovarian Development and Identify Sex in Fish

ZHI-GANG SHEN, HONG YAO, LIANG GUO, XIAO-XIA LI, AND HAN-PING WANG


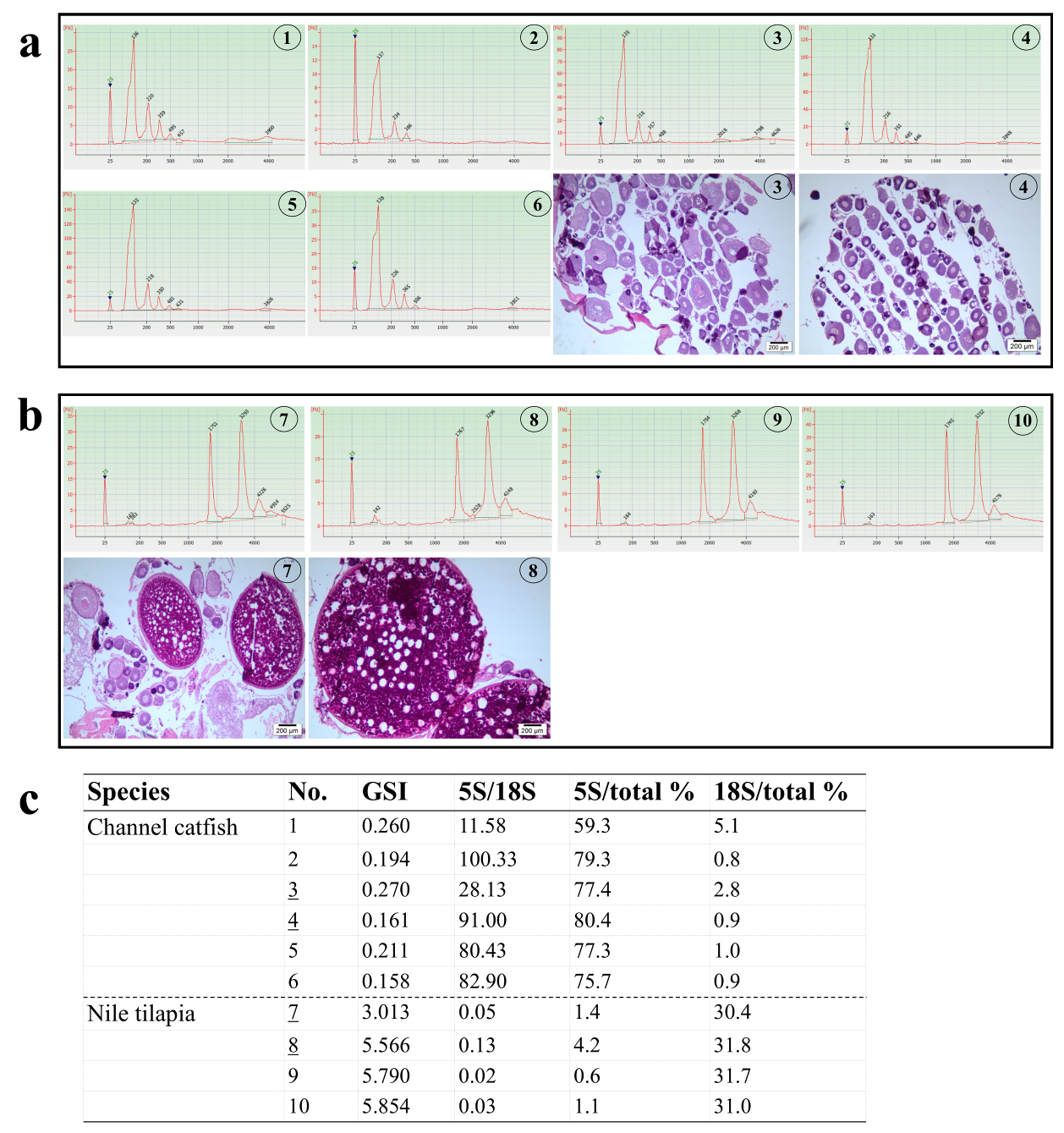


Figure S1. **Total RNA electropherograms of ovary samples in Channel catfish and Nile tilapia and corresponding rRNA profiling**. **a**, electropherograms for 6 ovary samples and histology for 2 ovary samples in Channel catfish; **b**, electropherograms for 4 ovary samples and histology for 2 ovary samples in Nile tilapia; **c**, corresponding rRNA profiling for the total 10 samples. GSI, gonadosomatic index. 5S/18S, 5S and 18S rRNA ratio; 5S/total %, percentage of 5S rRNA relative to total RNA; 18S/total %, percentage of 18S rRNA relative to total RNA.


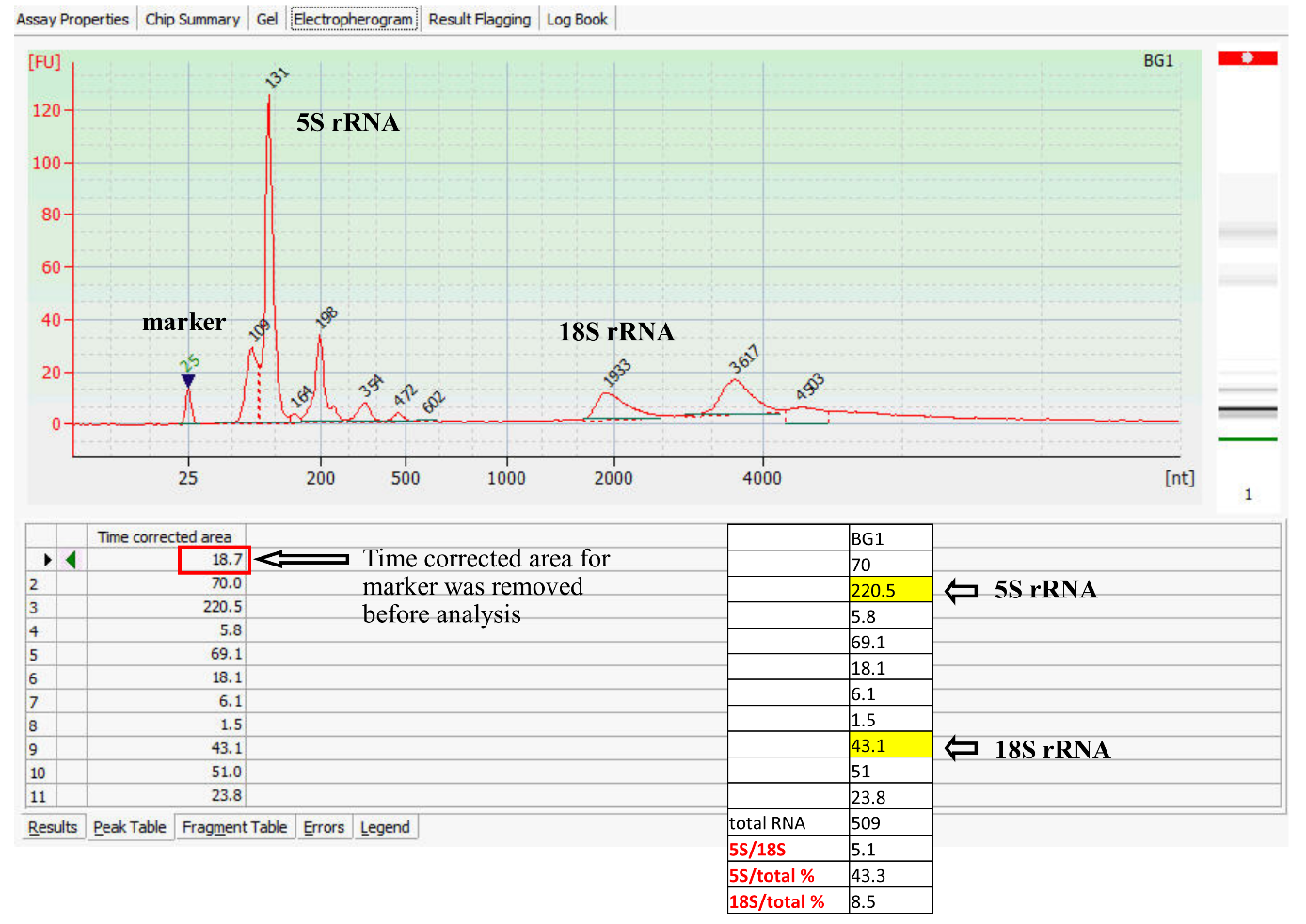


Figure S2 **An example of ribosome RNA profiling using Agilent Bioanalyzer 2100 Expert software (free version)**. Three steps are summarized: **1**. Identification of 5S and 18S rRNA peaks. These two rRNA could be easily identified according to their size and abundance. Value on the top of each peak indicate size (nucleotide, nt) for each peak. **2**. Copy time correct area to Excel sheet and remove time corrected area for marker. **3**. Calculate 5S-18S rRNA ratio, 5S rRNA percentage relative to total RNA content, and 18S rRNA percentage.
